# Correction: Role of the early secretory pathway in SARS-CoV-2 infection

**DOI:** 10.1083/jcb.20200600508132020c

**Published:** 2020-08-24

**Authors:** Daria Sicari, Aristotelis Chatziioannou, Theodoros Koutsandreas, Roberto Sitia, Eric Chevet

Vol. 219, No. 9 | 10.1083/jcb.202006005 | July 28, 2020

The initial published version of Figure 1 contained a topological error regarding virion budding, which has been corrected. The authors and editor apologize for any confusion. This error appears only in PDF versions downloaded on or before August 24, 2020.

**Figure 1:**
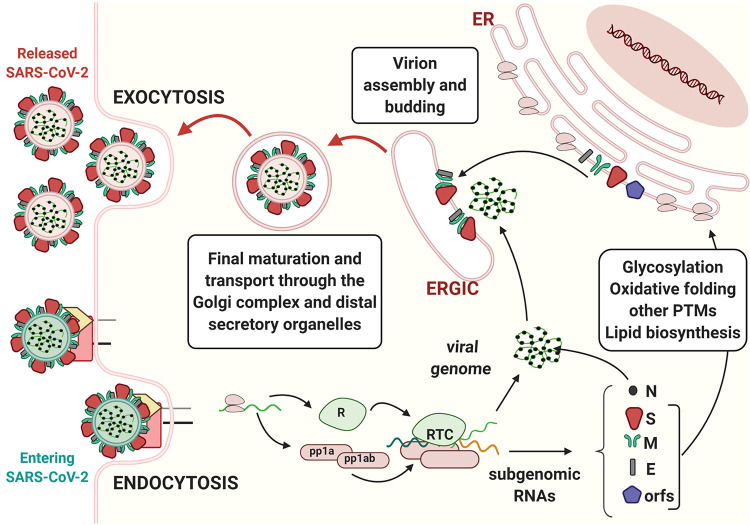


**The journey of SARS-CoV-2 in the host cell.** Coronavirus binds to cognate receptors on target cells via the spike proteins (dark red). This drives conformational changes that promote fusion of the virus with the host cell’s plasma membrane (entry by endocytosis and green membrane–containing virion, bottom left). In the cytoplasm, viral capsids are uncoated, and the viral RNA genome is translated, producing two poly-proteins (pp1a and pp1ab). These polypeptides are then proteolytically processed by both host and viral proteases, thereby generating nonstructural proteins (nsps) and leading to the formation of the replicase–polymerase complex (RTC). The latter is responsible for the replication of the viral genome and for the production of subgenomic RNAs, which are translated into the structural proteins nucleocapsid (N), spike (S), membrane (M), and envelope (E). In addition to these genomic elements shared by other CoVs, the SARS-CoV-2 genome also contains eight open reading frames (ORFs) that drive the production of accessory proteins. S, M, and E structural proteins and some accessory proteins are co-translationally translocated into the ER, where they undergo diverse post-translational modifications, including disulfide bond formation and N-linked glycosylation. Structural proteins concentrate in the ERGIC, where they assemble around the newly formed genome–nucleocapsid complexes. Mature virions are further modified (e.g., O-glycosylated) as they proceed through the Golgi complex and later stations of the secretory pathway before being released in the extracellular milieu (release by exocytosis and pink membrane–containing virions, top left). The membrane of the virus derives from the host cell, which synthetizes it in the ER.

